# Antimicrobial co-resistance patterns of gram-negative bacilli isolated from bloodstream infections: a longitudinal epidemiological study from 2002–2011

**DOI:** 10.1186/1471-2334-14-393

**Published:** 2014-10-12

**Authors:** Patrick HP Wong, Marcus von Krosigk, Diane L Roscoe, Tim TY Lau, Masoud Yousefi, William R Bowie

**Affiliations:** Division of Infectious Diseases, Department of Medicine, Faculty of Medicine, The University of British Columbia (UBC), 452D, Heather Pavilion East, VGH, 2733 Heather Street, Vancouver, BC V5Z 3J5 Canada; Faculty of Pharmaceutical Sciences, UBC, Vancouver, Canada; Department of Pathology and Laboratory Medicine, Faculty of Medicine, UBC, Vancouver, Canada; Division of Medical Microbiology and Infection Control, Vancouver General Hospital (VGH), Vancouver, Canada; Brain Research Centre, UBC, Vancouver, Canada

**Keywords:** Co-resistance, Multi-drug resistance, Gram-negative bacilli, Bloodstream infections, Antibiograms

## Abstract

**Background:**

Increasing multidrug resistance in gram-negative bacilli (GNB) infections poses a serious threat to public health. Few studies have analyzed co-resistance rates, defined as an antimicrobial susceptibility profile in a subset already resistant to one specific antibiotic. The epidemiologic and clinical utility of determining co-resistance rates are analyzed and discussed.

**Methods:**

A 10-year retrospective study from 2002–2011 of bloodstream infections with GNB were analyzed from three hospitals in Greater Vancouver, BC, Canada. Descriptive statistics were calculated for antimicrobial resistance and co-resistance. Statistical analysis further described temporal trends of antimicrobial resistance, correlations of resistance between combinations of antimicrobials, and temporal trends in co-resistance patterns.

**Results:**

The total number of unique blood stream isolates of GNB was 3280. Increasing resistance to individual antimicrobials was observed for *E. coli*, *K. pneumoniae*, *K. oxytoca*, *E. cloacae*, and *P. aeruginosa*. Ciprofloxacin resistance in *E. coli* peaked in 2006 at 40% and subsequently stabilized at 29% in 2011, corresponding to decreasing ciprofloxacin usage after 2007, as assessed by defined daily dose utilization data. High co-resistance rates were observed for ceftriaxone-resistant *E. coli* with ciprofloxacin (73%), ceftriaxone-resistant *K. pneumoniae* with trimethoprim-sulfamethoxazole (83%), ciprofloxacin-resistant *E. cloacae* with ticarcillin-clavulanate (91%), and piperacillin-tazobactam-resistant *P. aeruginosa* with ceftazidime (83%).

**Conclusions:**

Increasing antimicrobial resistance was demonstrated over the study period, which may partially be associated with antimicrobial consumption. The study of co-resistance rates in multidrug resistant GNB provides insight into the epidemiology of resistance acquisition, and may be used as a clinical tool to aid prescribing empiric antimicrobial therapy.

**Electronic supplementary material:**

The online version of this article (doi:10.1186/1471-2334-14-393) contains supplementary material, which is available to authorized users.

## Background

Gram-negative bacilli (GNB) are a significant cause of infection in community and nosocomial settings [1]. Besides inherent and chromosomally mediated mechanisms of resistance, the development of multidrug resistance in GNB is further facilitated by the acquisition of plasmids, integrons and transposons carrying resistance genes, which is typically a consequence of selective antimicrobial pressure exerted by prolonged antibiotic use [2]. It is also becoming increasingly common to find multiple resistance genes that are linked together, thus antibiotics from unrelated classes may contribute to the selective pressures and maintain the expression of these multidrug resistant genes [3, 4].

The Infectious Diseases Society of America views antimicrobial resistance as a serious threat to public health, patient safety and national security, and has published policy recommendations for the US Congress to address the rising rates of antibiotic resistance together with declining approvals of new antibiotics [5]. Pop-Vicas and D’Agata (2005) have emphasized the need to further expand our understanding of the dynamics of transmission of these multidrug resistant pathogens to determine whether it is related to cross transmission from humans to humans or antimicrobial selective pressures [6]. Kallen and Srinivasan (2010) have also highlighted the importance for ongoing surveillance of the incidence and epidemiology of multidrug resistant GNB [1].

Several studies have shown an increasing incidence of multidrug resistant GNB [7–9]. However, to our knowledge, few studies provide a comprehensive evaluation of co-resistance patterns with antimicrobial agents commonly used to treat GNB. Co-resistance is defined in this study as the antimicrobial susceptibility profile in a subset of isolates already resistant to a specific antibiotic, and provides a different means for monitoring multidrug resistance and displaying observed trends, such as increasing multidrug resistance in *P. aeruginosa* that are resistant to ciprofloxacin [10–12]. The study of co-resistance can thus be quite broad and not limited to isolates that are known to harbour multidrug resistance, such as those with ESBLs. From a clinical perspective, co-resistance should be taken into consideration when prescribing empiric therapy for patients being treated for specific GNB where local antimicrobial resistance rates are significant, and in patients who have been exposed to prior courses of antimicrobial agents.

The aim of our study is to examine the antimicrobial co-resistance patterns of GNB bloodstream isolates over a 10-year time period in order to document changes in susceptibility patterns, and to identify any potential causes when significant changes occur. The utility of the information gathered would be applicable in clinical practice when tailoring empiric antibiotic treatment.

## Methods

We conducted a 10-year retrospective study from Jan 2002 to Dec 2011 to quantify the trends of resistance and co-resistance patterns in GNB isolated from bloodstream infections at Vancouver Coastal Health (VCH), which includes Vancouver General Hospital (VGH) (a 950-bed tertiary care teaching hospital with 21,000 admissions per year), Richmond Hospital (a 175-bed community hospital), and Lion's Gate Hospital (a 268-bed community hospital). The study was approved by the University of British Columbia Clinical Research Ethics Board and by the Vancouver Coastal Health Research Institute.

Utilizing the VCH laboratory information system, Sunquest®, which stores laboratory data in the Sunset® database, a report was generated to identify all positive blood cultures with GNB from 2002–2011. Further refinement was performed through computer programming to include only one bacterial isolate of the same identification (genus and species) and susceptibility pattern per patient per calendar year in the analysis. The first unique patient isolate from the first admission during each study year was used in cases where multiple isolates of the same genus, species and susceptibility pattern were identified. When multiple cultures taken from the same patient had the same GNB identified but with any difference in susceptibility patterns, these were considered unique isolates and were included in the analysis. The Sunquest® system started to incorporate data for Richmond Hospital in April 2005 and Lion's Gate Hospital in August 2007. Annual review of the antimicrobial susceptibility patterns from the individual hospitals in this study do not differ significantly from each other and inclusion of additional sites over the study period was not felt to influence results.

Identification and susceptibility testing were performed using commercially available automated systems [BD Phoenix™ (Sep 2009 to study end date) or Siemens MicroScan® (2002 to Sept 2009) systems]. Quality control measures were implemented during the changeover of automated systems in 2009 for validation and verification to ensure that the results on both systems were concordant within acceptable limits. Antibiotic susceptibility testing was performed and interpreted using guidelines and interpretive breakpoints for susceptible, intermediate, and resistant categories established by the Clinical and Laboratory Standards Institute (CLSI). All antimicrobial susceptibility results that fell into the intermediate category were presumed to be resistant for the purposes of this study. Guidelines for susceptibility breakpoints for cephalosporins were changed after 2010, but the laboratory did not change to the new guidelines since the panels for the commercial system in use did not have concentration wells that were low enough to allow interpretation at the lower breakpoints [13].

The data collected was extrapolated from Sunset® into a Microsoft Access® database. Queries and reports of individual antimicrobial resistance and co-resistance patterns were generated to supply descriptive statistics for each specific study year and pathogen. Pharmacy data on inpatient antimicrobial consumption at VGH in the form of defined daily doses (DDDs) were compared against antimicrobial resistance results. Statistical analysis was performed to determine linear-to-linear temporal trends of individual antimicrobial resistance patterns. In addition, Pearson correlation coefficients were determined for associations between resistant combinations of antimicrobial agents, and Pearson Chi-square test was performed to assess for temporal trends in co-resistance patterns. P values of ≤0.05 were used for statistical significance in all cases.

## Results

**Figure 1:**
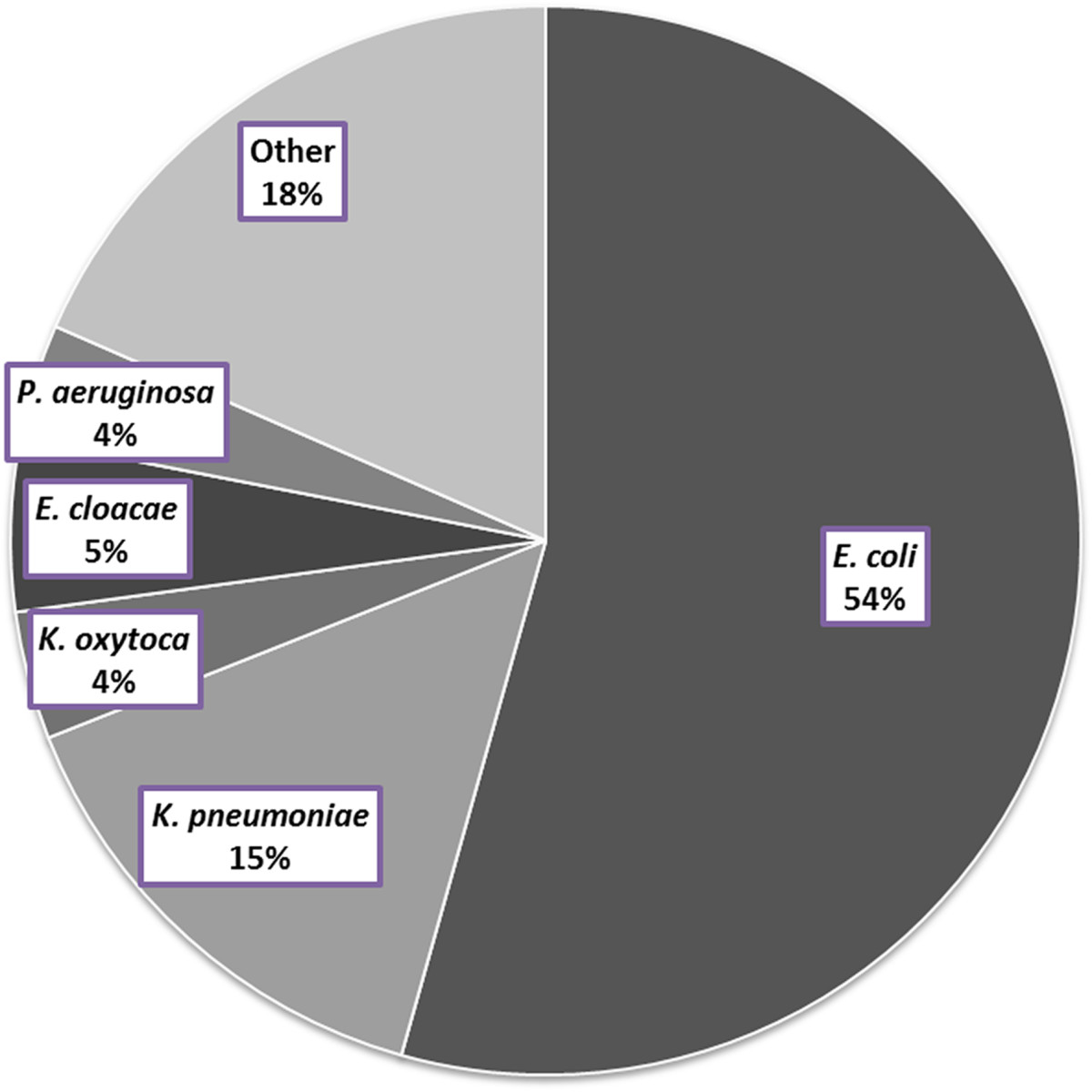
**Distribution of gram-negative bacilli isolated from bloodstream isolates from 2002–2011.** The total number of unique bloodstream isolates during the study period was 3280.

**Figure 2:**
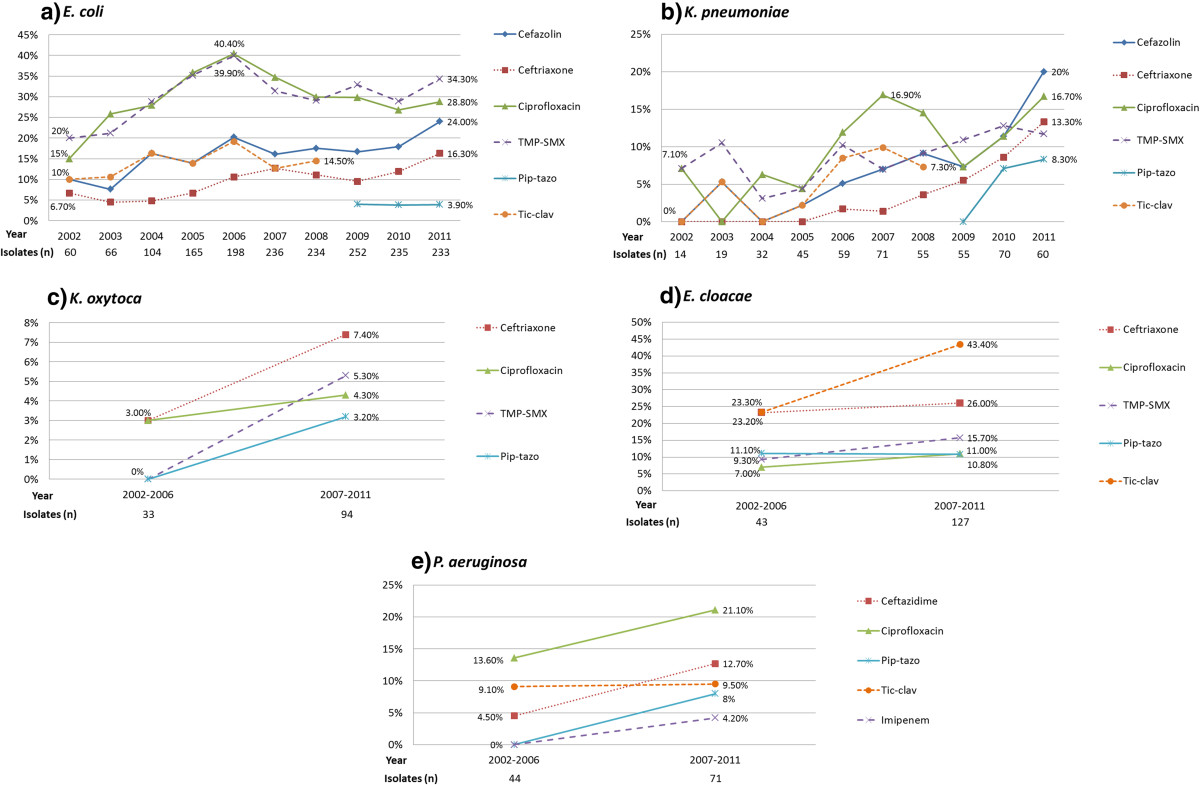
**Temporal patterns of resistance to selected antimicrobial agents for a)** ***E. coli*** **, b)** ***K. pneumoniae*** **, c)** ***K. oxytoca*** **, d)** ***E. cloacae*** **, and e)** ***P. aeruginosa*** **.** Antimicrobial resistance rates were analyzed yearly for *E. coli* and *K. pneumoniae*, and combined into two time groups of 2002–06 and 2007–11 for *K. oxytoca*, *E. cloacae*, and *P. aeruginosa* due to lower isolate numbers. Number of isolates for each GNB is listed below the corresponding year or time groups. Y-axis represents resistance in percentage. Note: Pip-tazo is piperacillin-tazobactam, TMP-SMX is trimethoprim-sulfamethoxazole, Tic-clav is ticarcillin-clavulanate.

The total number of unique blood stream isolates of GNB was 3,280 over the 10-year study period, ranging annually from 109 (2002) to 463 (2010). The five most common GNB isolated were *Escherichia coli*, *Klebsiella pneumoniae*, *Klebsiella oxytoca*, *Enterobacter cloacae*, and *Pseudomonas aeruginosa*, and the proportion of each is illustrated in Figure 1. The temporal trends of resistance to commonly used antibiotics for each of these organisms are illustrated by line graphs in Figure 2.

**Figure 3:**
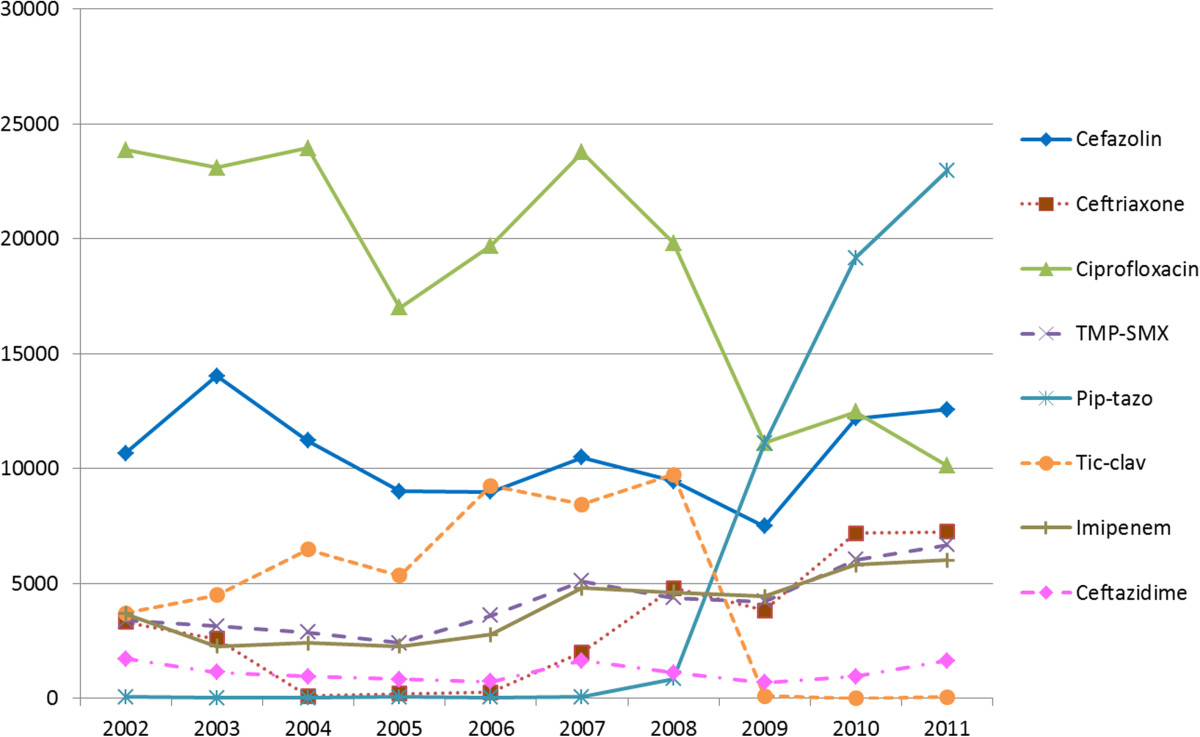
**Inpatient antimicrobial utilization at Vancouver General Hospital in defined daily dose (DDD) by calendar year.** DDD is listed on Y-axis. Note: Pip-tazo is piperacillin-tazobactam, TMP-SMX is trimethoprim-sulfamethoxazole, Tic-clav is ticarcillin-clavulanate.

For *E. coli* (Figure 2a), overall trends of increasing resistance to cefazolin and ceftriaxone were observed over time. Resistance to piperacillin-tazobactam remained stable at 4% from 2009 to 2011; it replaced ticarcillin-clavulanate on the hospital formulary in 2009. Resistance to ciprofloxacin and trimethroprim-sulfamethoxazole (TMP-SMX) both peaked during 2006 at roughly 40% and stabilized at lower levels of 29% and 34% respectively in 2011. Comparing this with antimicrobial consumption data (Figure 3), use of ciprofloxacin peaked in 2007 at 23,800 DDD and decreased to 10,100 DDD in 2011, whereas TMP-SMX consumption peaked in 2007 at 5,100 DDD, dropped to 4,200 DDD in 2009, and has increased back to 6,700 DDD in 2011. A subgroup analysis of ESBL E. *coli* (total of 54 isolates in 2010 and 2011) revealed the following resistance rates: ciprofloxacin (83%), piperacillin-tazobactam (17%), TMP-SMX (76%), gentamicin (52%), ceftriaxone (100%), and imipenem (0%). In contrast, non-ESBL *E. coli* (total of 414 isolates in 2010 and 2011) had lower resistance rates: ciprofloxacin (21%), piperacillin-tazobactam (2%), TMP-SMX (26%), gentamicin (8%), ceftriaxone (3%), and imipenem (0%).

For *K. pneumoniae* (Figure 2b), overall trends of increasing resistance were observed with cefazolin, ceftriaxone and piperacillin-tazobactam. Subgroup analysis for ESBL *K. pneumoniae* (total of 12 isolates in 2010 and 2011) revealed the following resistance rates: ciprofloxacin (50%), piperacillin-tazobactam (42%), TMP-SMX (50%), gentamicin (42%), ceftriaxone (100%), and imipenem (0%). In contrast, non-ESBL *K. pneumoniae* (total of 118 isolates in 2010 and 2011) had lower resistance rates: ciprofloxacin (10%), piperacillin-tazobactam (4%), TMP-SMX (8%), gentamicin (2%), ceftriaxone (2%), and imipenem (1%).

For *K. oxytoca* (Figure 2c), increasing rates of resistance to ceftriaxone, ciprofloxacin, TMP-SMX, and piperacillin-tazobactam were observed. For *E. cloacae* (Figure 2d), resistance rates increased with ceftriaxone, TMP-SMX, and ciprofloxacin, but remained relatively stable with piperacillin-tazobactam. For *P. aeruginosa* (Figure 2e), resistance rates to ceftazidime, ciprofloxacin, and piperacillin-tazobactam all increased. *P. aeruginosa* resistance to imipenem was 4.2% during 2007–2011 (from a baseline of 0% in 2002–2006).

Chi-square trend tests were performed to determine which temporal patterns of antimicrobial resistance fit into a linear-by-linear association model. Linear-by-linear trends were identified with cefazolin resistance in *E. coli* (X^2^ = 9.062, p < 0.003), ceftriaxone resistance in *E. coli* (X^2^ = 13.070, p < 0.001), cefazolin resistance in *K. pneumoniae* (X^2^ = 15.183, p < 0.001), ceftriaxone resistance in *K. pneumoniae* (X^2^ = 18.066, p < 0.001), piperacillin-tazobactam resistance in *K. pneumoniae* (X^2^ = 5.485, p < 0.019), and cefazolin resistance in *K. oxytoca* (X^2^ = 4.329, p < 0.037). Other temporal patterns that approached significance in a linear-by-linear association model include ciprofloxacin resistance in *K. pneumoniae* (X^2^ = 3.656, p = 0.056), TMP-SMX resistance in *K. oxytoca* (X^2^ = 3.576, p = 0.059), ticarcillin-clavulanate resistance in *K. pneumoniae* (X^2^ = 3.716, p = 0.054), and ceftazidime resistance in *P. aeruginosa* (X^2^ = 3.213, p = 0.073). The temporal trend models for *E. coli* resistant to ciprofloxacin and SMX-TMP are curvilinear with calculated departure of linearity as a chi-square for ciprofloxacin is 22.611 on 8df (p < 0.0039), and for TMP-SMX is 15.31 on 8df (p = 0.053).

The second part of the analysis involved looking at correlation patterns of resistance between various pairs of antibiotics, which is commonly reported in other publications but does not provide information regarding co-resistance as defined in this study. In *E. coli* (Table 1a), high correlations of resistance were identified between several combinations of antimicrobial agents including piperacillin-tazobactam with ciprofloxacin (r = 0.973), ticarcillin-clavulanate with cefazolin (r = 0.885), TMP-SMX with ciprofloxacin (r = 0.871), ticarcillin-clavulanate with TMP-SMX (r = 0.839), and ceftriaxone with cefazolin (r = 0.808). In *K. pneumoniae* (Table 1b), highest correlations of resistance were seen between piperacillin-tazobactam with ceftriaxone (r = 0.982), ceftriaxone with cefazolin (r = 0.935), and ticarcillin-clavulanate with cefazolin (r = 0.893). In *K. oxytoca* (Table 1c), highest correlations of resistance were between ciprofloxacin with ceftriaxone (r = 0.952), ticarcillin-clavulanate with ceftriaxone (r = 0.773), and ticarcillin-clavulanate with ciprofloxacin (r = 0.773). In *E. cloacae* (Table 1d), highest correlations of resistance were observed between ticarcillin-clavulanate with ceftriaxone (r = 0.944), piperacillin-tazobactam with ceftriaxone (r = 0.927), and ticarcillin-clavulanate with TMP-SMX (r = 0.781). Finally, in *P. aeruginosa* (Table 1e), resistance to ticarcillin-clavulanate was highly correlated with ceftazidime resistance (r = 0.888).

**Table 1:** **Pearson correlation matrixes showing Pearson correlation coefficients (r) of resistance between pairs of antibiotics for a)** ***E. coli*** **, b)** ***K. pneumonia*** **, c)** ***K. oxytoca*** **, d)** ***E. cloacae*** **, e)** ***P. aeruginosa***

a) Pearson correlation matrix (r) for ***E. coli***						
	Cefazolin	Ceftriaxone	Ciprofloxacin	TMP-SMX	Pip-tazo	Tic-clav
Cefazolin (n = 1783)						
Ceftriaxone (n = 1783)	**0.808**					
Ciprofloxacin (n = 1783)	0.487	0.290				
TMP-SMX (n = 1783)	**0.770**	0.503	**0.871**			
Pip-tazo (n = 846)	0.702	0.623	**0.973**	***0.865***		
Tic-clav (n = 1063)	**0.885**	0.299	***0.733***	**0.839**	nd	
**b) Pearson correlation matrix (r) for** ***K. pneumoniae***						
	**Cefazolin**	**Ceftriaxone**	**Ciprofloxacin**	**TMP-SMX**	**Pip-tazo**	**Tic-clav**
Cefazolin (n = 480)						
Ceftriaxone (n = 480)	**0.935**					
Ciprofloxacin (n = 480)	***0.630***	0.566				
TMP-SMX (n = 480)	**0.719**	**0.689**	0.274			
Pip-tazo (n = 163)	0.917	**0.982**	0.886	0.778		
Tic-clav (n = 295)	**0.893**	0.677	0.648	0.635	nd	
**c) Pearson correlation matrix (r) for** ***K. oxytoca***						
	**Cefazolin**	**Ceftriaxone**	**Ciprofloxacin**	**TMP-SMX**	**Pip-tazo**	**Tic-clav**
Cefazolin (n = 127)						
Ceftriaxone (n = 127)	0.576					
Ciprofloxacin (n = 127)	***0.619***	**0.952**				
TMP-SMX (n = 127)	*−* ***0.598***	−0.005	−0.184			
Pip-tazo (n = 71)	0.011	−0.222	−0.349	−0.174		
Tic-clav (n = 65)	0.649	**0.773**	**0.773**	0.013	nd	
**d) Pearson correlation matrix (r) for** ***E. cloacae***						
	**Ceftriaxone**	**Ciprofloxacin**	**TMP-SMX**	**Pip-tazo**	**Tic-clav**	
Ceftriaxone (n = 170)						
Ciprofloxacin (n = 170)	**0.688**					
TMP-SMX (n = 170)	***0.611***	0.172				
Pip-tazo (n = 83)	**0.927**	0.542	0.781			
Tic-clav (n = 96)	**0.944**	0.613	**0.781**	nd		
**e) Pearson correlation matrix (r) for** ***P. aeruginosa***						
	**Ceftazidime**	**Ciprofloxacin**	**Pip-tazo**	**Tic-clav**		
Ceftazidime (n = 115)						
Ciprofloxacin (n = 115)	−0.457					
Pip-tazo (n = 60)	0.177	0.225				
Tic-clav (n = 65)	**0.888**	−0.100	nd			

Number of isolates (n) included in each matrix is listed for each row. Pearson coefficients (r) which are bolded represent pairs with p values ≤ 0.05, while those that are bolded and italicized trended towards significance with p values > 0.05 ≤ 0.07. Note: TMP-SMX is trimethoprim-sulfamethoxazole, Pip-tazo is piperacillin-tazobactam, Tic-clav is ticarcillin-clavulanate; nd is not determined as isolates did not consistently have both Pip-tazo and Tic-clav susceptibilities reported.

The final part of our analysis involved selecting out a group of isolates that were resistant to one specific antimicrobial agent and then examining that group's antimicrobial susceptibility profile–the co-resistance pattern. In *E. coli* (Table 2a), those that are resistant to ceftriaxone have a 73% probability of also exhibiting resistance to ciprofloxacin. In contrast, for those that are resistant to ciprofloxacin, only 25% are also resistant to ceftriaxone. Other high co-resistance rates found in *E. coli* were piperacillin-tazobactam-resistant strains that are co-resistant with TMP-SMX (77%), piperacillin-tazobactam-resistant strains that are co-resistant with ciprofloxacin (71%), and ceftriaxone-resistant strains that are co-resistant with TMP-SMX (69%). To assess temporal patterns, Pearson Chi-Square tests were performed to analyze whether co-resistance rates remained stable or changed significantly over time. Over time comparing the periods of 2002–2006 and 2007–2011, the rate of ceftriaxone-resistant *E. coli* that are co-resistant with ciprofloxacin has been stable around 73% (X^2^ = 0.005, p = 0.941) suggesting no significant change. However, the rate of ciprofloxacin-resistant *E. coli* that are co-resistant with ceftriaxone has increased from 16% to 30% (X^2^ = 12.103, p < 0.001).

**Table 2:** **Summary of antimicrobial co**-**resistance rates determined for a**) ***E*** *.* ***coli***, **b**) ***K*** *.* ***pneumoniae***, **c**) ***K*** *.* ***oxytoca***, **d**) ***E*** *.* ***cloacae***, **and e**) ***P*** *.* ***aeruginosa***

a) ***E. coli***						
No. of isolates (n)	Resistance to	Co-resistance rate with antibiotic (%)				
2002-06		Ceftriaxone	Ciprofloxacin	Pip-tazo	TMP-SMX	Tic-clav
44	Ceftriaxone		**73**	14	**59**	**59**
194	Ciprofloxacin	16		8	**62**	31
3	Pip-tazo	33	**67**		**67**	nd
193	TMP-SMX	13	**63**	8		31
91	Tic-clav	29	**67**	nd	**66**	
**2007-11**						
146	Ceftriaxone		**73**	18	**72**	48
357	Ciprofloxacin	30		10	**58**	26
28	Pip-tazo	**57**	**71**		**79**	nd
373	TMP-SMX	28	**56**	10		27
64	Tic-clav	42	**61**	nd	**61**	
**2002**-**11**						
190	Ceftriaxone		**73**	18	**69**	**53**
551	Ciprofloxacin	25		10	**60**	29
31	Pip-tazo	**55**	**71**		**77**	nd
566	TMP-SMX	23	**58**	9		30
155	Tic-clav	34	**65**	nd	**64**	
**b)** ***K. pneumoniae***						
**No. of isolates (n)**	**Resistance to**	**Co-resistance rate with antibiotic (%)**				
**2002-2011**		**Ceftriaxone**	**Ciprofloxacin**	**Pip-tazo**	**TMP-SMX**	**Tic-clav**
20	Ceftriaxone		**60**	35	**83**	**53**
54	Ciprofloxacin	22		22	46	29
10	Pip-tazo	**60**	**50**		40	nd
44	TMP-SMX	30	**57**	16		37
19	Tic-clav	26	**58**	nd	**53**	
**c)** ***K. oxytoca***						
**No. of isolates (n)**	**Resistance to**	**Co-resistance rate with antibiotic (%)**				
**2002-2011**		**Ceftriaxone**	**Ciprofloxacin**	**Pip-tazo**	**TMP-SMX**	**Tic-clav**
8	Ceftriaxone		38	20	25	**50**
5	Ciprofloxacin	**60**		0	20	25
2	Pip-tazo	**50**	0		0	nd
5	TMP-SMX	40	20	0		**100**
3	Tic-clav	**67**	33	nd	33	
**d)** ***E. cloacae***						
**No. of isolates (n)**	**Resistance to**	**Co-resistance rate with antibiotic (%)**				
**2002-2011**		**Ceftriaxone**	**Ciprofloxacin**	**Pip-tazo**	**TMP-SMX**	**Tic-clav**
43	Ceftriaxone		26	47	37	**81**
17	Ciprofloxacin	**65**		17	**59**	**91**
9	Pip-tazo	**100**	11		11	nd
24	TMP-SMX	**67**	42	6		**80**
33	Tic-clav	**67**	30	nd	24	
**e)** ***P. aeruginosa***						
**No. of isolates (n)**	**Resistance to**	**Co-resistance rate with antibiotic (%)**				
**2002-2011**		**Ceftazidime**	**Ciprofloxacin**	**Pip-tazo**	**Imipenem**	**Gentamicin**
11	Ceftazidime		45	**50**	27	9
22	Ciprofloxacin	23		24	23	23
6	Pip-tazo	**83**	**67**		33	33
13	Imipenem	23	38	18		23
9	Gentamicin	11	**56**	29	33	

Co-resistance rates were analyzed as a summary of isolates from 2002–2011 except for *E. coli*, where it was possible to analyze two time periods from 2002–06 and 2007–11 due to higher isolate numbers. Co-resistance rates are rounded to the nearest percent, and those that are ≥50% are highlighted in bold font. Note: Pip-tazo is piperacillin-tazobactam, TMP-SMX is trimethoprim-sulfamethoxazole, Tic-clav is ticarcillin-clavulanate; nd is not determined as isolates did not consistently have both Pip-tazo and Tic-clav susceptibilities reported.

For *K. pneumoniae* (Table 2b), high co-resistance rates were seen with ceftriaxone-resistant strains that are co-resistant with TMP-SMX (83%), ceftriaxone-resistant strains that are co-resistant with ciprofloxacin (60%), and piperacillin-tazobactam-resistant strains that are co-resistant with ceftriaxone (60%). For *K. oxytoca* (Table 2c), high co-resistance was seen between ciprofloxacin-resistant strains with ceftriaxone (60%), however it is important to note that only 5 isolates of *K. oxytoca* were analyzed.

For *E. cloacae* (Table 2d), highest co-resistance rates were observed with piperacillin-tazobactam-resistant strains that are co-resistant with ceftriaxone (100%), ciprofloxacin-resistant strains that are co-resistant with ticarcillin-clavulanate (91%), ceftriaxone-resistant strains that are co-resistant with ticarcillin-clavulanate (81%), and TMP-SMX resistant strains that are co-resistant with ticarcillin-clavulanate (80%).

In *P. aeruginosa* (Table 2e), high co-resistance rates were observed with piperacillin-tazobactam-resistant strains that are co-resistant with ceftazidime (83%), and piperacillin-tazobactam-resistant strains that are co-resistant with ciprofloxacin (67%). It has been previously observed that *P. aeruginosa* resistant to fluoroquinolones are often associated with resistance to other antibiotic classes [10, 12]. Interestingly, our results do not suggest a particularly high co-resistance profile in ciprofloxacin-resistant *P. aeruginosa* with co-resistance rates of 23-24% with gentamicin, imipenem, ceftazidime, and piperacillin-tazobactam.

## Discussion

To our knowledge, this is one of the largest studies comprehensively evaluating antimicrobial co-resistance in GNB isolated from bloodstream infections. In the first part of our study, an unanticipated finding in our results was that both ciprofloxacin and TMP-SMX resistance peaked in 2006 for *E. coli*, and has subsequently decreased and stabilized.

Our experience at VGH is that there had previously been heavy consumption of ciprofloxacin during the time period when the resistance peaked, which suggests this selective pressure may have contributed to this observation. We reviewed consumption data of antibiotics at VGH based on inpatient utilization data (Figure 3), and determined that the (DDD) of ciprofloxacin was stable at 23,000-24,000 from 2002–2004, decreasing to 17,000 in 2005, increasing back to 23,800 in 2007, and has since decreased to 10,100 in 2011. In comparison for ceftriaxone utilization, the DDD has continually increased from 3,300 to 7,200 from 2002 to 2011. The utilization data supports that resistance rates of *E. coli* to ciprofloxacin and ceftriaxone correlate with consumption patterns of antibiotics during that period, which peaked in 2007 and 2011, respectively (Figure 2a). The DDD for TMP-SMX is also of interest in that there were two consumption peaks – 5,100 in 2007 and 6,700 in 2011, which again correlates with resistance peaks in our *E. coli* resistance data and provides further support that resistance patterns may be associated with antimicrobial consumption (Figure 2a). As most of the GNB isolates were collected from VGH, the changes in resistance patterns are most attributable to the antimicrobial consumption at that site. In general, the antimicrobial consumption data collected from VGH is representative of the usage at the other two smaller hospitals sites.

When looking at antimicrobial resistance patterns of *E. coli* in the community collected by BC Biomedical Laboratories (45 community-based patient services centres in Greater Vancouver, BC), we observe a significant jump in the resistance rates of *E. coli* to ciprofloxacin between 2002 and 2007 (10% to 22%) [14]. It is noted from the BC Centre for Disease Control publication that there was no *E. coli* data published for the years 2003–2006 because antibiograms were not produced when the resistance rates remained relatively similar, suggesting that the jump in resistance may have occurred in 2007. However, unlike our data after 2007, the level of resistance remained relatively stable until a further increasing trend in 2010 (26%) and 2011 (27%) [14]. One explanation for this observed difference is that data from BC Biomedical Laboratories is reflective of an outpatient-based setting, where ciprofloxacin continues to be heavily utilized for urinary tract infections. Fortunately due to concerns regarding resistance, the 2010 Infectious Disease Society of America (IDSA) guideline for uncomplicated cystitis now recommends fluoroquinolone antibiotics as alternative agents only when other urinary tract infection therapies cannot be used [15]. As seen by our utilization data at VGH, in the hospital setting where patients are more ill in general, physicians are now prescribing less ciprofloxacin as empiric antimicrobial therapy because of the high rates of resistance in both inpatient and outpatient settings.

A further clinical utility of determining co-resistance rates is to aid in the selection of empiric antimicrobial coverage. For example, patients with suspected urinary tract infection are often started on TMP-SMX while pending culture results. However, if the patient deteriorates and is not responding clinically, one would have to decide which antibiotic to use to broaden the coverage. In the periods from 2002–2006, co-resistance of *E. coli* to ceftriaxone was only 13%, so it may have been appropriate to switch to this antibiotic. However, co-resistance of *E. coli* to ceftriaxone was 28% in 2007–2011, thus it may be more reasonable based on our results to switch to piperacillin-tazobactam which has a co-resistance rate of only 10%. This is of particular importance with the advances in bacterial identification techniques, such as Matrix Assisted Laser Desorption Ionization – Time of Flight (MALDI-TOF) where bacterial species are identified on average 1.45 days earlier than traditional methods [16]. The limitation of this technique is that susceptibility testing is still required, further separating the time gap between identification of bacteria to reporting of susceptibility results. Knowledge of local antimicrobial resistance and co-resistance rates becomes more critical in prescribing empiric antimicrobial therapy during this period. In addition, for initial empiric therapy in critically ill patients one may want to consider combination antimicrobial coverage, especially in patients at high risk for acquiring multidrug resistant organisms. With co-resistance data, one can calculate resistant rates of using various combinations of empiric antimicrobial treatment. For example, 15% of *P. aeruginosa* isolates are resistant to gentamicin (2011), and gentamicin-resistant *P. aeruginosa* that are co-resistant with piperacillin-tazobactam, imipenem and ceftazidime are 29, 33 and 11%, respectively. The resistance rates of utilizing combination gentamicin with piperacillin-tazobactam (0.15 × 0.29), imipenem (0.15 × 0.33) or ceftazidime (0.15 × 0.11) in initial management are 4.4, 5.0, and 1.6%, respectively. This is an interesting finding as one may not intuitively expect the lower resistance rates with the utilization of combination therapy with ceftazidime versus imipenem.

A major strength of our study is that the isolates were included from a diverse area within the Greater Vancouver Regional District, which included the cities of Vancouver, Richmond, and North Vancouver. The inclusion criteria were stringent to ensure that duplicate samples from the same patient were not included in the analysis. Our use of only bloodstream isolates allowed us to reliably negate effects from other possible ‘contaminant’ specimens, as GNB isolated from blood samples are almost invariably indicative of true infections. Patients with positive blood cultures are generally more acutely ill and would benefit most from the clinical utility of using co-resistance data in the selection of an empiric antimicrobial regimen. One limitation of this study is that we were unable to determine whether these bloodstream infections were community- or nosocomially-acquired. Due to the low number of isolates for *K. oxytoca*, *E. cloacae*, and *P. aeruginosa*, the resistance data had to be grouped into two time points, which reduced the ability to determine annual variations that may have occurred. Another limitation was that we were unaware of whether a patient received any recent or concurrent antimicrobial therapies when blood cultures were drawn, which may have yielded additional information regarding the incidence of failed empiric therapy. In addition, we did not explicitly determine the source of bacteremia, and so are unable to tell if resistance strains and patterns were more commonly associated with certain types of infections. Although we classified all intermediate susceptible isolates as being resistant which would seemingly elevate the overall resistance rates, this methodology is consistent with clinical practice in which an antimicrobial agent with intermediate susceptibility would generally not be used if there were other effective alternatives. Overall, the incidence of intermediate susceptible isolates was generally low during the study period ranging from 0% to 2% for most of the antibiotic agents.

Besides ongoing surveillance of co-resistance patterns, one potential application for the future would be to look at co-resistance patterns in various medical services and ward locations. It has been proposed that unit-based antibiograms and combination antibiograms may be more useful than national or hospital-specific antibiograms [17]. Unit-based antibiograms refers to cumulative antibiotic susceptibility reports for patients in a particular ward over a specified period of time, whereas combination antibiograms provide information on percent susceptibility to other antibiotics if it is resistant to one particular antibiotic [17], or essentially the co-resistance rate that we investigated in this study. Unit-based combination antibiograms can be considered for certain wards, such as the leukemia/bone marrow transplant ward where patients can be critically ill, as this population may benefit from combination antibiotic empiric coverage depending on local susceptibility patterns. The ability of MALDI-TOF technology to expedite identification of pathogens emphasizes the need and importance of having accurate and up to date antimicrobial susceptibility data.

## Conclusions

In summary, increasing antimicrobial resistance was observed for several GNB over a 10-year period, and may partially be associated with antimicrobial consumption. The study of co-resistance rates in multidrug resistant GNB may provide further insight into the epidemiology of resistance acquisition. Further applications for co-resistance data include its utility as a clinical tool to aid in the prescription of empiric antimicrobial therapy.

## Authors’ original submitted files for images

Below are the links to the authors’ original submitted files for images. Authors’ original file for figure 1 Authors’ original file for figure 2 Authors’ original file for figure 3 Authors’ original file for figure 4 Authors’ original file for figure 5 Authors’ original file for figure 6 Authors’ original file for figure 7
