# Supplementary figures and images for: Antimicrobial co-resistance patterns of gram-negative bacilli isolated from bloodstream infections: a longitudinal epidemiological study from 2002–2011
Source: BMC Infect Dis. 2014 Oct 12;14:393. doi: 10.1186/1471-2334-14-393 (PMC4287581; doi:10.1186/1471-2334-14-393)

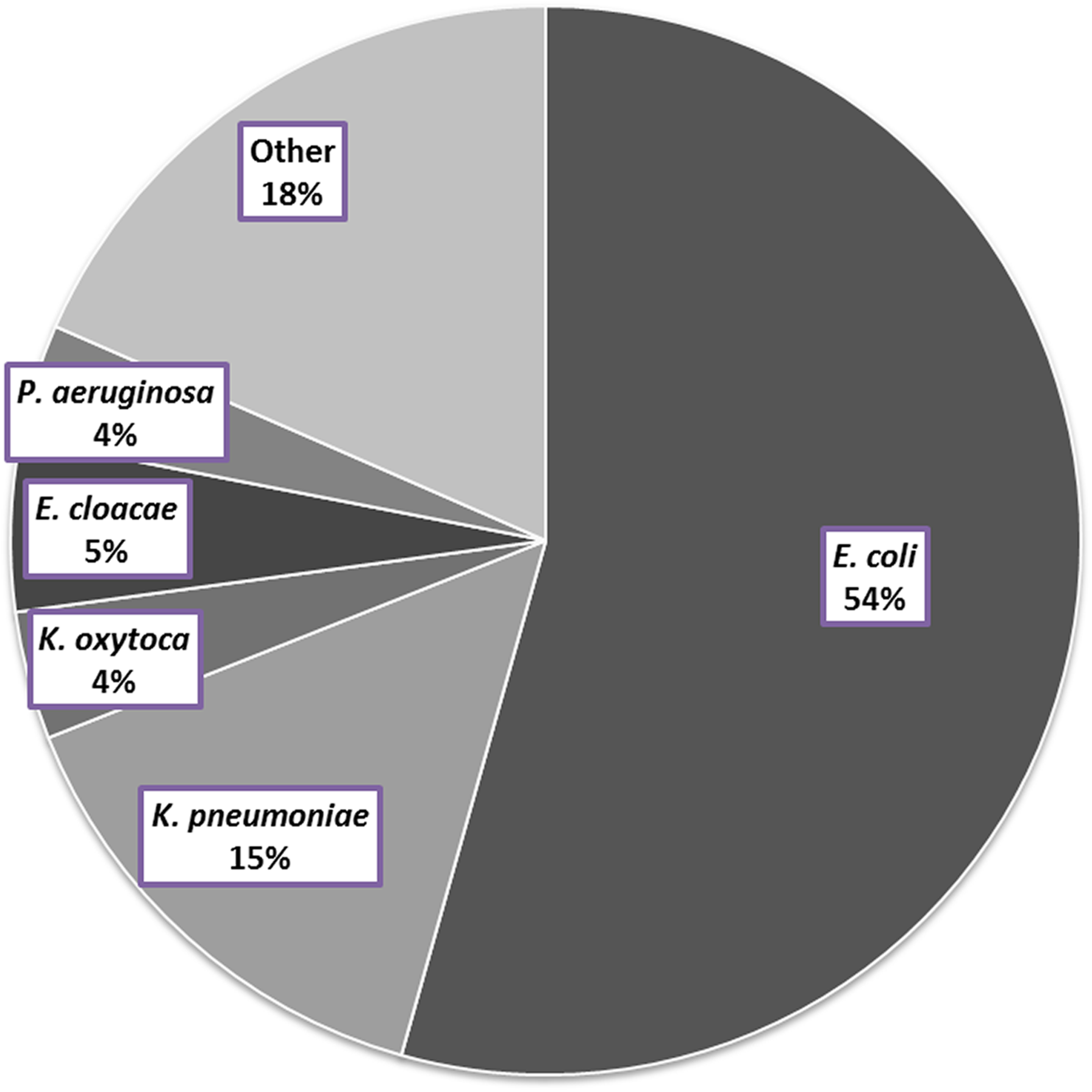

Supplement: Supplementary file 1 — Authors’ original file for figure 1 [file 12879_2014_3852_MOESM1_ESM.tiff]

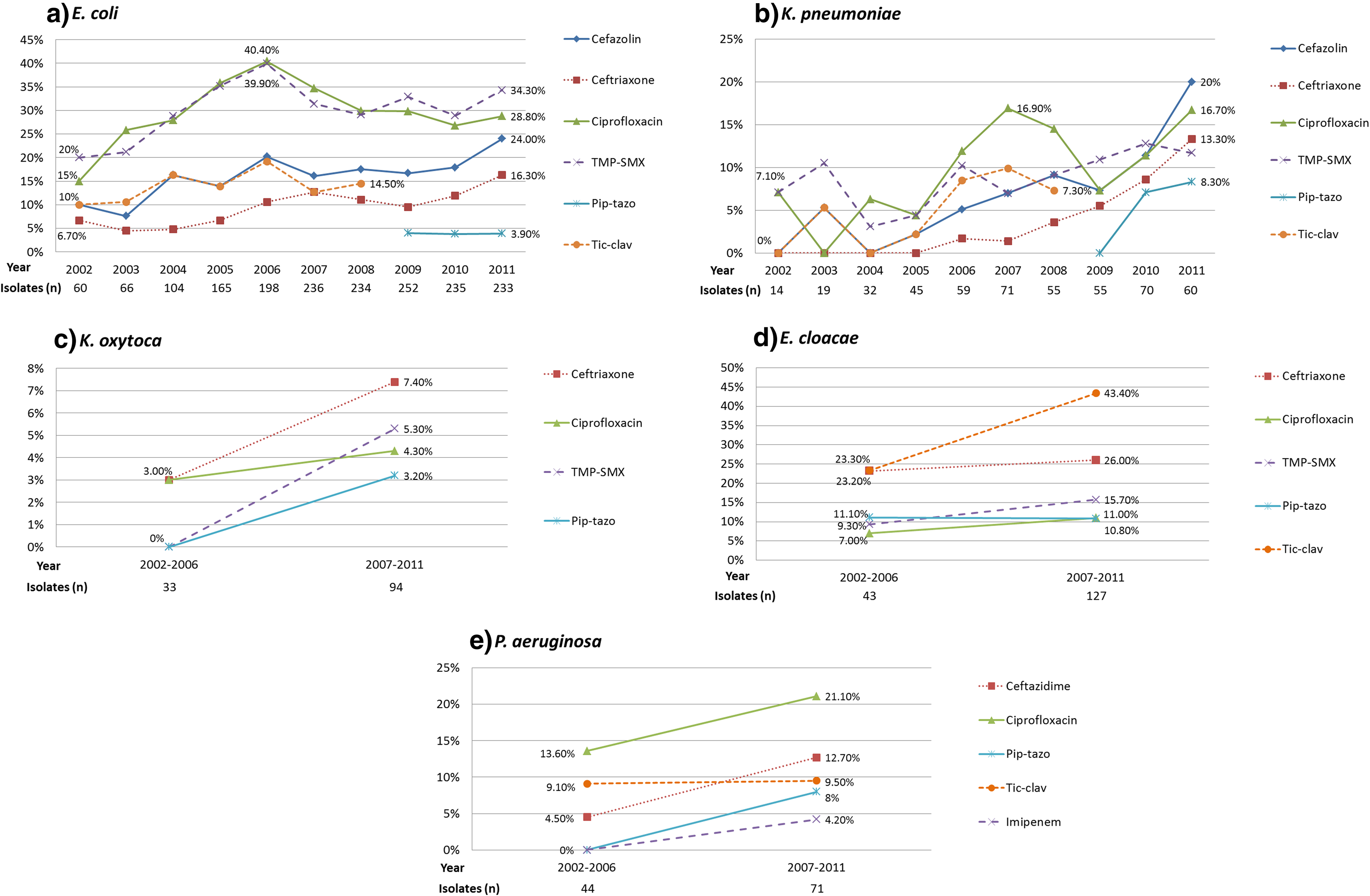

Supplement: Supplementary file 2 — Authors’ original file for figure 2 [file 12879_2014_3852_MOESM2_ESM.tiff]

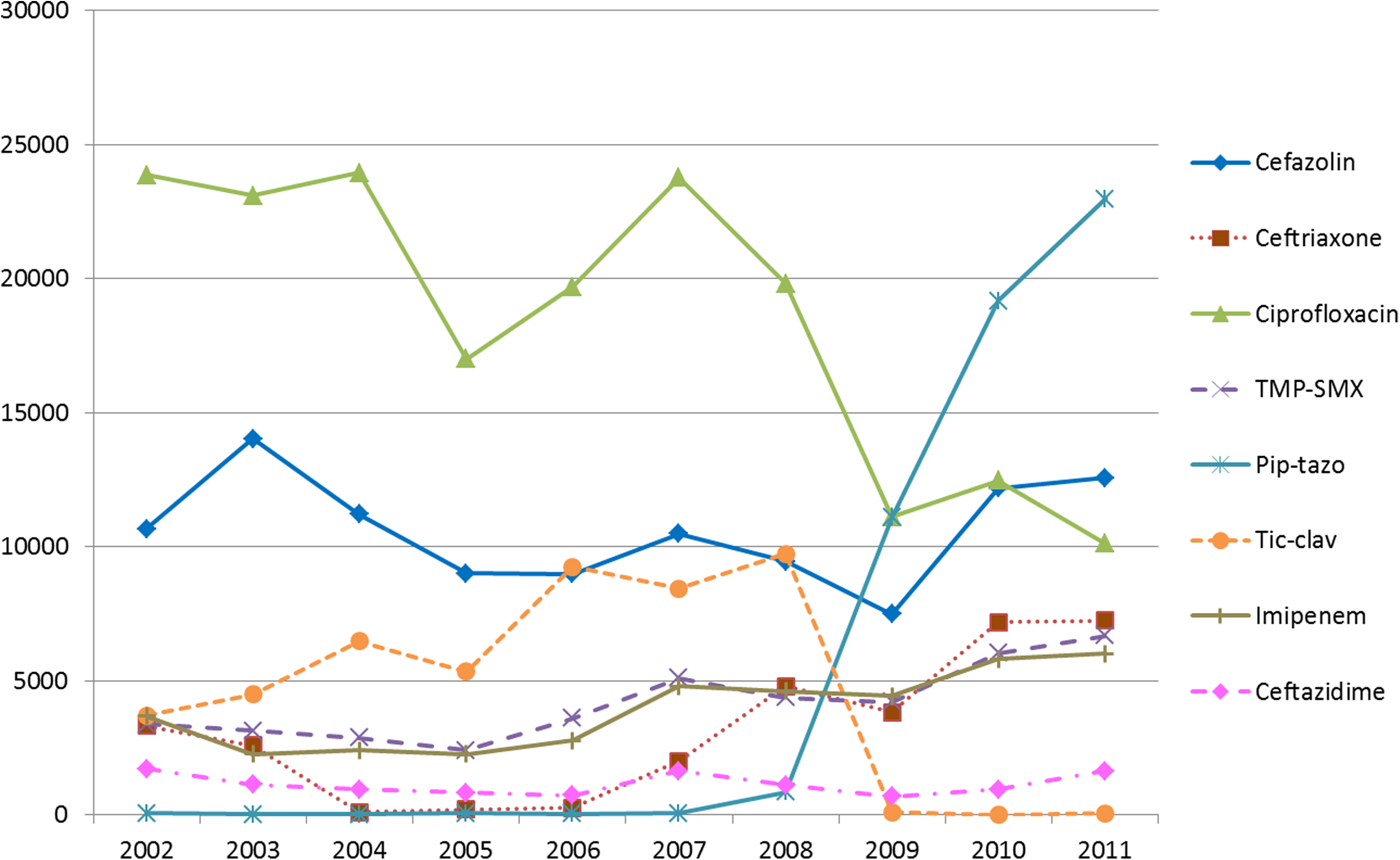

Supplement: Supplementary file 3 — Authors’ original file for figure 3 [file 12879_2014_3852_MOESM3_ESM.tiff]

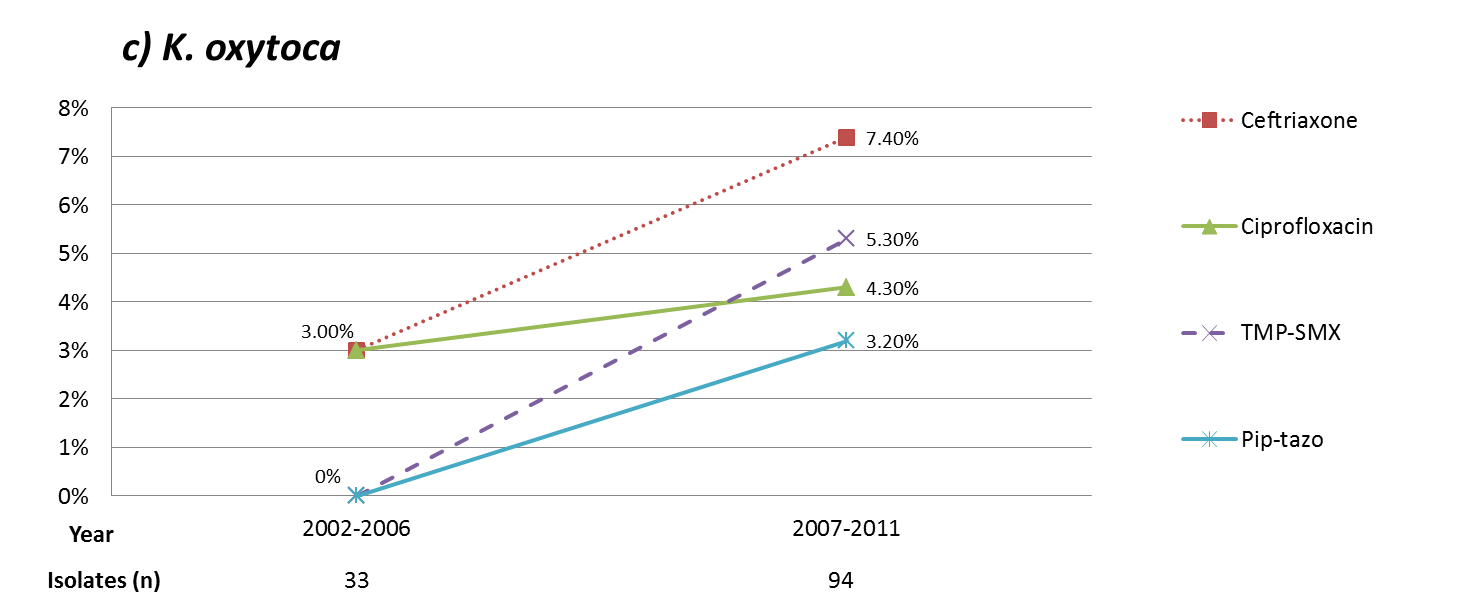

Supplement: Supplementary file 4 — Authors’ original file for figure 4 [file 12879_2014_3852_MOESM4_ESM.png]

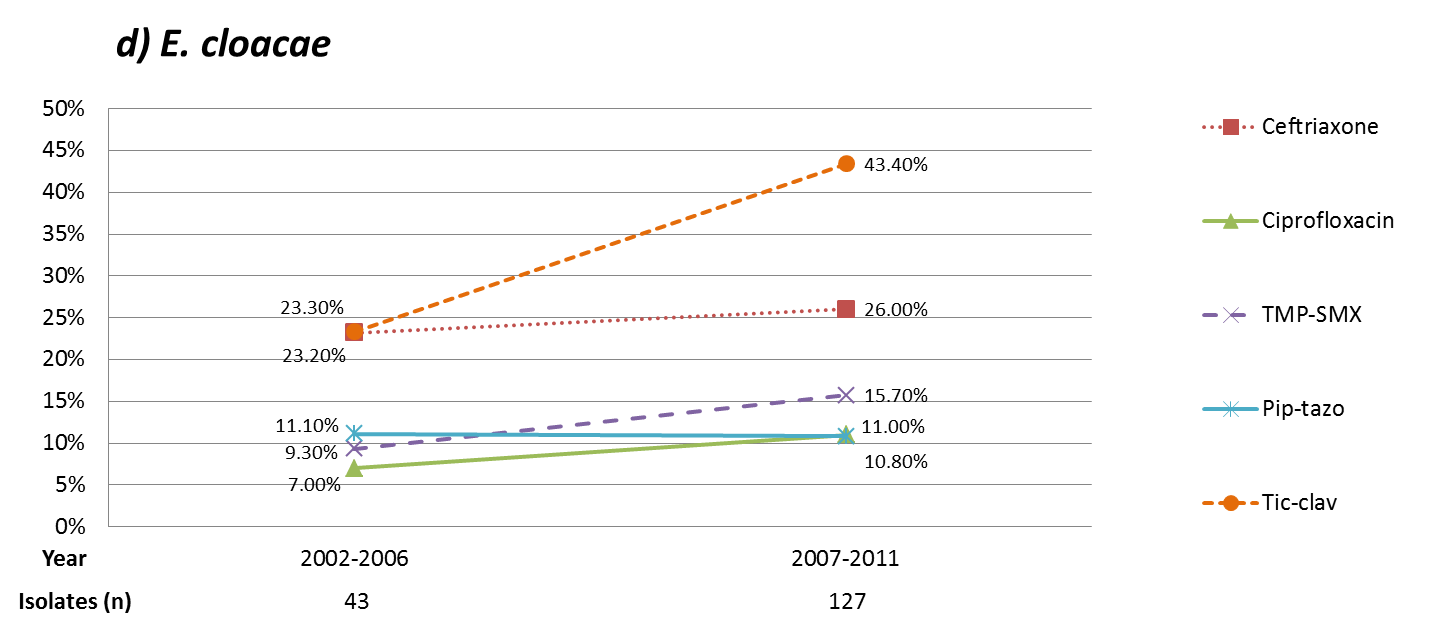

Supplement: Supplementary file 5 — Authors’ original file for figure 5 [file 12879_2014_3852_MOESM5_ESM.png]

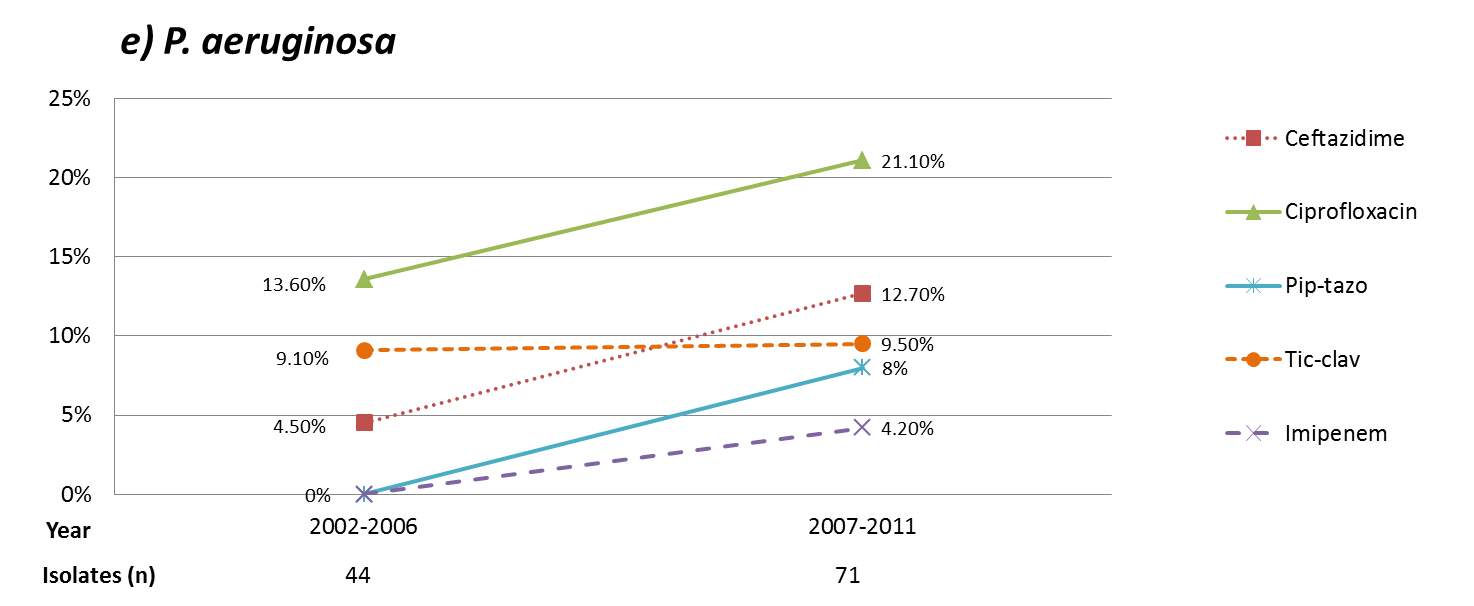

Supplement: Supplementary file 6 — Authors’ original file for figure 6 [file 12879_2014_3852_MOESM6_ESM.png]

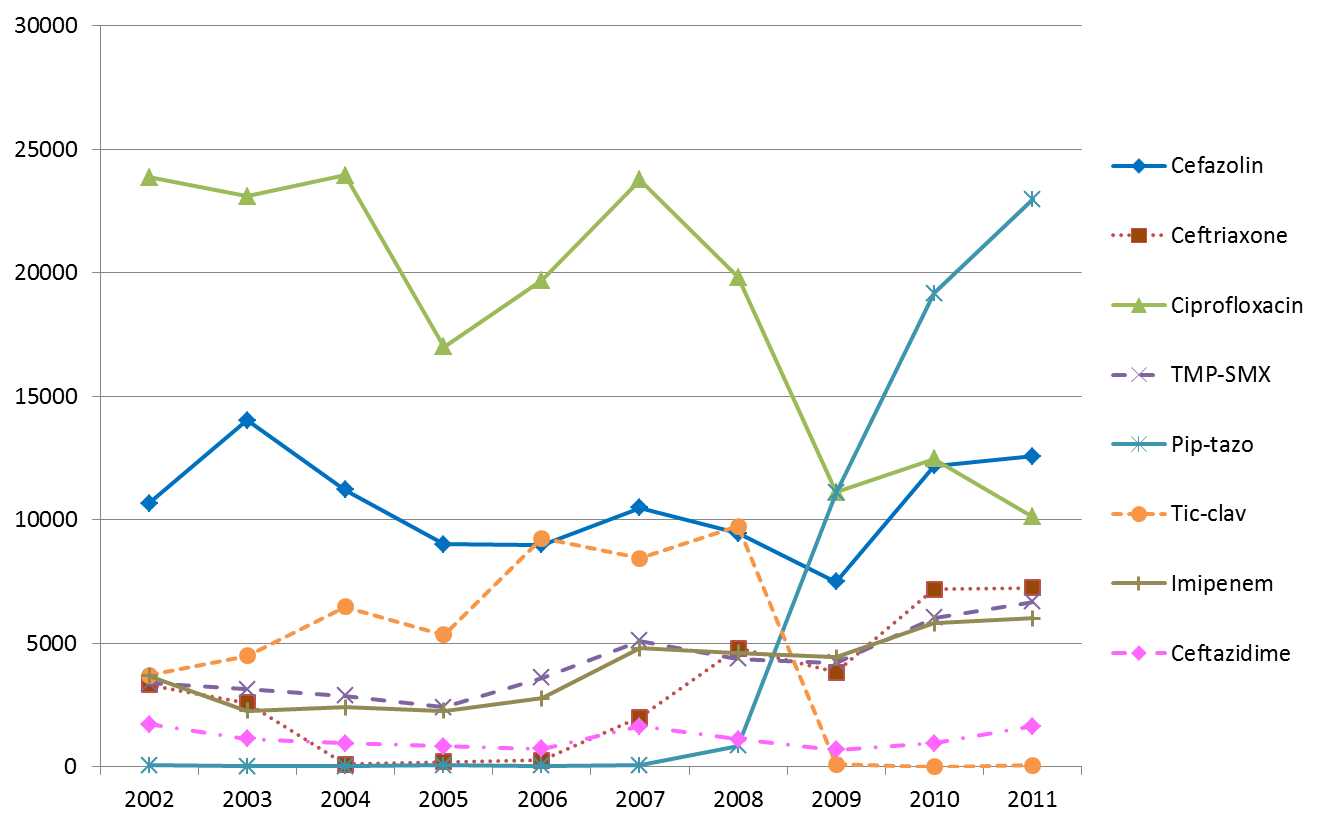

Supplement: Supplementary file 7 — Authors’ original file for figure 7 [file 12879_2014_3852_MOESM7_ESM.png]
